# Modifications of the charge–discharge behaviour of Fe_2_(MoO_4_)_3_ in all-solid-state lithium-ion batteries†

**DOI:** 10.1039/d4ra03058c

**Published:** 2024-06-06

**Authors:** Yuta Kimura, Shintaro Kobayashi, Shogo Kawaguchi, Koji Ohara, Yasuhiro Suzuki, Takashi Nakamura, Yasutoshi Iriyama, Koji Amezawa

**Affiliations:** a Institute of Multidisciplinary Research for Advanced Materials, Tohoku University 2-1-1, Katahira, Aoba-Ku Sendai 980-8577 Japan yuta.kimura.b2@tohoku.ac.jp; b Japan Synchrotron Radiation Research Institute 1-1-1, Koto, Sayo-cho Sayo-gun Hyogo 679-5198 Japan; c Faculty of Materials for Energy, Shimane University 1060, Nishikawatsu-cho Matsue Shimane 690-0823 Japan; d Graduate School of Engineering, Nagoya University Furo, Chikusa Nagoya Aichi 464–8603 Japan; e Institute of Materials and Systems for Sustainability, Nagoya University Furo, Chikusa Nagoya Aichi 464–8603 Japan

## Abstract

The solidification of lithium-ion batteries (LIBs) by replacing liquid electrolytes with solid electrolytes enables the development of a new class of LIBs, namely all-solid-state lithium-ion batteries (ASSLIBs), with improved safety and energy density. Such battery solidification can greatly influence the properties of battery components, as exemplified by a recent report suggesting that the (dis)charge behaviour of Fe_2_(MoO_4_)_3_ (FMO), a promising two-phase electrode material, differs on solid electrolytes compared to liquid electrolytes. However, its underlying mechanism remains unclear. Here we examined the (de)lithiation behaviour of FMO thin films on solid electrolytes using *operando* synchrotron X-ray diffraction (XRD) to gain insights into the influence of the solidification on the (dis)charge mechanism of electrode materials. The XRD results revealed that FMO on solid electrolytes exhibits a monotonic peak shift over a wide capacity range, accompanied by a temporary peak broadening. This suggests that FMO possesses an expanded solid-solution reaction region and a narrower two-phase reaction region in solidified batteries compared to liquid-based LIBs. The altered (dis)charge behavior was suggested to be thermodynamically driven, as it remained largely unchanged with varying rates and under open circuit conditions. Qualitative analysis considering stress-induced variations in Gibbs free energy curves demonstrated that external stress, potentially caused by the constraint of chemo-mechanical expansion, can thermodynamically narrow the two-phase region when the chemical expansion coefficients of the two phases of FMO differ. These findings highlight the significant impact of the battery solidification on electrode material properties, emphasizing the importance of considering these unique issues in the design of ASSLIBs.

## Abstract 2

*Operando* X-ray diffraction measurements revealed that Fe_2_(MO_4_)_3_ electrode thin films on solid electrolytes possess an expanded solid-solution reaction region and narrower two-phase reaction region compared to those in liquid electrolytes.

## Graphical abstract

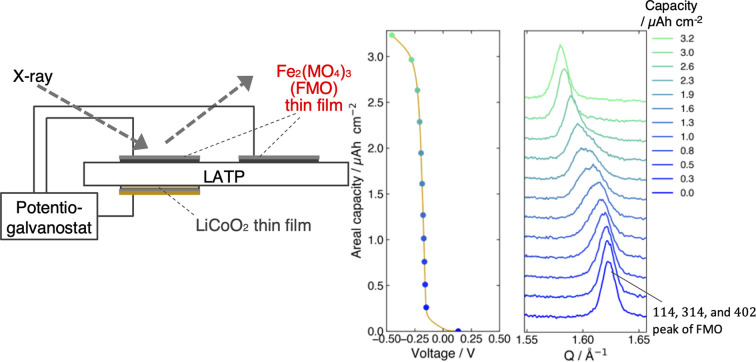

## Introduction

Electrochemical energy storage systems, represented by lithium-ion batteries (LIBs), are employed as power sources for a wide range of portable electronic devices, including smartphones and laptops, and have become an indispensable technology in our daily lives.^1,2^ Recently, the demand for LIBs is further increasing due to their growing use in larger-scale, high-current applications such as electric vehicles and smart grids.^3,4^ Although these applications require further enhancements in the capacity, power output, cycle life, and safety of LIBs, it is pointed out that the energy density of LIBs is approaching its physicochemical limit.^5^ Furthermore, the use of organic liquid electrolytes poses intrinsic safety risks for current LIBs, including leakage, thermal runaway, and explosion hazards.^6^

To address these issues, the solidification of LIBs by replacing liquid electrolytes with solid electrolytes has attracted significant attention.^7–9^ The use of inflammable solid electrolytes eliminates the aforementioned risks associated with liquid electrolytes, greatly improving battery safety. Moreover, solid electrolytes potentially allow the use of Li metal anodes, which would substantially increase the energy density.^10^ Therefore, fully solidified LIBs, namely, all-solid-state lithium-ion batteries (ASSLIBs), are regarded as a next-generation electrochemical energy storage technology and have been the subject of extensive research and development efforts in recent years.^11,12^

Through these ASSLIB studies, unique phenomena arising from the solidification of LIBs have been reported. Both theoretical^13,14^ and experimental^15,16^ studies reported that the space charge layers, which cause the local inhomogeneous distribution of ions and electrons, forms at the interfaces between the solid battery components.^17^ Such an imbalance in charge carrier concentrations at the interface can lead to increased interfacial resistance, potentially degrading battery performance.^18,19^ Furthermore, in ASSLIBs, the strain induced in electrode materials by lithiation/delithiation, *i.e.*, the chemo-mechanical strain,^20^ is rigidly constrained at the solid–solid interfaces, resulting in significant stress in the battery components.^21,22^ Such large stress not only causes mechanical degradation in ASSLIBs,^23–25^ but also has been reported to greatly influence various properties of battery components, including electrical conductivity,^26,27^ electrode potential,^20,28^ reaction mechanisms,^29,30^ and phase equilibria.^31,32^ As described above, battery components in ASSLIBs are subjected to environments that differ from those in liquid-based LIBs; consequently, their material properties may deviate from those in their liquid-based counterparts.

One example of this is the modulation of the (dis)charge behaviour of Fe_2_(MoO_4_)_3_ (FMO) on solid electrolytes. FMO is an anti-NASICON-type polyanion compound that can reversibly intercalate lithium and sodium ions, making it a promising electrode material for lithium-ion and sodium-ion batteries.^33–40^ Particularly, thin-film FMO with shorter ion diffusion distances exhibits superior rate capability and cyclability,^36,40,41^ and is reported to demonstrate stable (dis)charge cycling in ASSLIBs.^40,42^ In liquid electrolytes, the lithiation/delithiation reaction of FMO proceeds *via* a typical two-phase reaction between FMO and Li_2_FMO, as confirmed by electrochemical measurements^37,38^ and synchrotron X-ray diffraction (XRD) measurements.^37^ Consequently, its (dis)charge curve exhibits a distinct potential plateau in liquid electrolytes.^38^ On the other hand, a recent study has reported that when FMO is deposited as a thin film on a solid electrolyte, its (dis)charge behaviour differs from that previously reported in liquid electrolytes. Specifically, the potential plateau during the (dis)charge becomes less pronounced, exhibiting a gradual potential variation instead.^40^ This implies that the lithiation/delithiation mechanism of FMO can be modulated on a solid electrolyte. If the use of solid electrolytes causes the modulations of the lithiation/delithiation behaviour of the electrode materials, investigating the underlying mechanisms is important for the proper design of ASSLIBs. Therefore, in this work, we examined the lithiation/delithiation behaviour of FMO thin films deposited on solid electrolytes using *operando* synchrotron XRD, aiming to gain insights into the influence of the solidification of LIBs on the (dis)charge mechanisms of electrode materials.

## Experimental

### Configuration and fabrication of ASSLIB cells

**Fig. 1:**
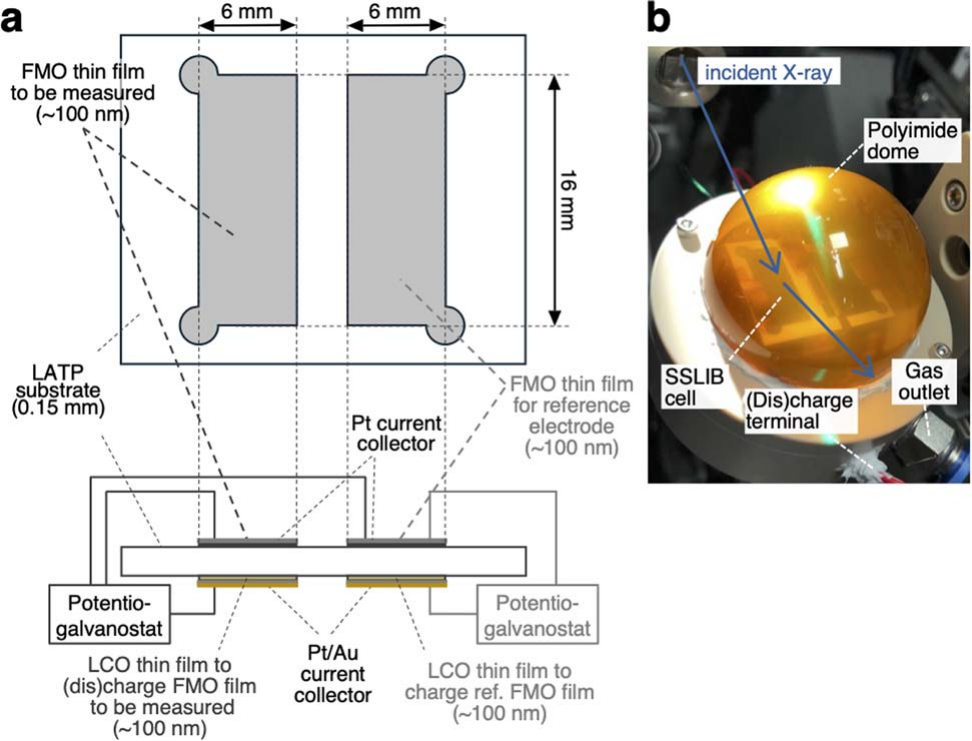
(a) Schematic of the ASSLIB cell and (b) picture of the sample holder employed in this work.

Fig. 1a depicts the configuration of the ASSLIB cell employed in this work. Two FMO thin films, each with an area of approximately 16 × 6 mm^2^ and a thickness of around 100 nm, were deposited on a Li_1+*x*_Al_*x*_Ti_2−*x*_(PO_4_)_3_ (LATP) (Ohara Inc. Japan) substrate with a thickness of about 150 μm. On the opposite side of the substrate, two LiCoO_2_ (LCO) thin films with nearly identical areas and thicknesses to the FMO films were deposited. LATP has a reduction threshold of the electrochemical window of approximately 2.2 V *vs.* Li^+^/Li,^43^ precluding the use of Li metal as a counter electrode. On the other hand, our previous work has demonstrated that employing a LCO thin film as a counter electrode enables the construction of ASSLIBs with relatively high cyclability.^40^ Therefore, in this study, we utilized the LCO thin films as the counter electrode, following our established approach. Furthermore, a Pt thin film with a thickness of approximately 30 nm was deposited on top of the FMO films for current collection, while Pt and Au thin films with a total thickness of around 100 nm were deposited on the LCO films for the same purpose. The deposition conditions for the FMO and LCO thin films were consistent with our previous report.^40^ The thickness of these films was measured using a stylus surface profilometer (Dektak 6M, Veeco Instruments Inc., USA). The cross-sectional SEM image and X-ray absorption near edge structure (XANES) spectra of the FMO thin film are included in ESI 1.† Prior to the XRD measurements, one of the FMO thin films was electrochemically lithiated to half of its theoretical capacity (3.3 μAh cm^−2^)^38,40^ by charging it as the negative electrode, with the LCO thin film on the opposite side of the substrate serving as the positive electrode. At this capacity, FMO is known to exhibit a stable electrode potential of approximately 3 V *vs.* Li^+^/Li. Thus, we employed this half-lithiated FMO thin film as a reference electrode in the XRD measurements. For the XRD measurements, a three-electrode cell was configured with another FMO thin film as the negative electrode, the remaining LCO thin film as the positive electrode, and the half-lithiated FMO thin film as the reference electrode. The FMO thin film was (dis)charged (Fe_2_(MO_4_)_3_ + 2Li^+^ + 2e^−^ ⇆ Li_2_Fe_2_(MO_4_)_3_) with the cut-off voltages of −0.45 and 0.35 V *vs.* the reference FMO electrode, and at (dis)charging current densities ranging from 0.51 to 51 μA cm^−2^, corresponding to C-rates of approximately 0.15C to 15C.

### *Operando* synchrotron XRD measurements of FMO thin films

*Operando* synchrotron XRD measurements of the FMO thin film during (dis)charging were performed at the BL02B2 beamline of SPring-8 in Japan. To prevent exposure of the FMO and LCO thin films to moisture in the air during (dis)charging, the sample was placed in a sample holder equipped with a polyimide dome, as shown in Fig. 1b, and 100% He was flowed inside the dome. X-rays with an energy of 15.5 keV and a beam size of 1 mm × 20 μm were incident on the FMO thin film from outside the dome at an incident angle of 0.5°. The XRD profiles of the FMO thin film during (dis)charging were then collected using a high-resolution one-dimensional solid-state detector (MYTHEN).^44^ The exposure time was set to 60 seconds for measurements at (dis)charging rates of 0.4C and below, 30 seconds for measurements at 1C and 2C (dis)charging rates, and 10 seconds for measurements at higher rates.

## Results and discussion

### XRD patterns of the pristine FMO thin film and that after lithiation

**Fig. 2:**
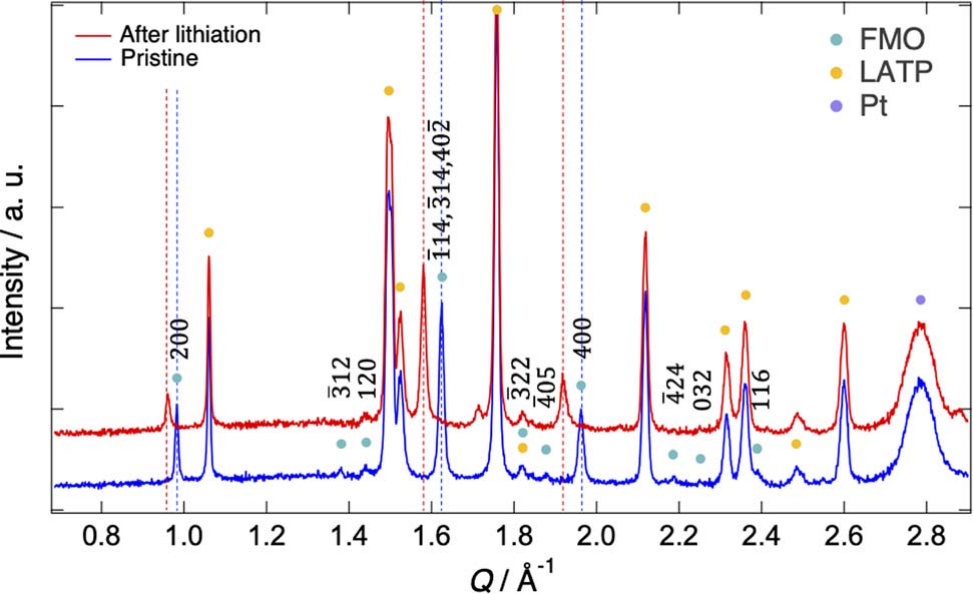
XRD patterns of the pristine FMO thin film and that after lithiation.

Fig. 2 shows the XRD patterns of the pristine FMO thin film and that after lithiation (XRD pattern after completing all measurements is shown in ESI 2†). The XRD patterns exhibited peaks corresponding to the FMO thin film, as well as those of the LATP substrate and Pt current collector. The peaks of the FMO thin film agreed with the *P*2_1_/*a* monoclinic FMO,^45^ and exhibited polycrystalline nature. On the other hand, the *h*00 peaks were more prominent compared to that of the powder XRD pattern of FMO, indicating a localized *a*-axis orientation of the FMO thin film on the LATP substrate, in agreement with a previous report.^40^ After lithiation, while no changes were observed in the peaks of the LATP substrate and Pt current collector, the positions of the FMO peaks, particularly the 200, 400, 1̄14, 3̄14, and 402̄ peaks, noticeably shifted to lower *Q* values, consistent with those in the literature for the lithiation of FMO in liquid electrolytes.^37^ In the following analysis, we focused on the main peak (1̄14, 3̄14, and 402̄ peak) of FMO, which exhibited the most significant changes during lithiation, to track the lithiation/delithiation reaction of the FMO thin film on the solid electrolyte.

### Lithiation/delithiation behaviour of the FMO thin film under constant current (dis)charging

**Fig. 3:**
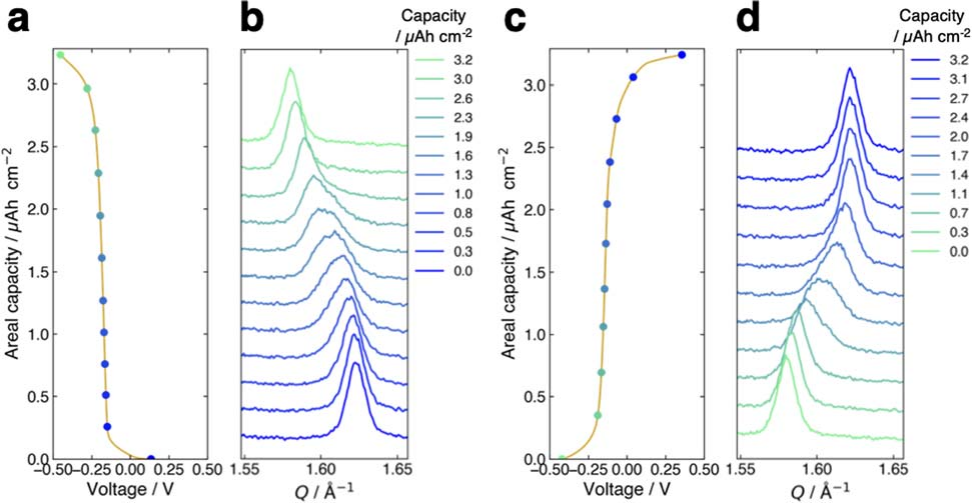
(a) Voltage curve and (b) main peak position variation of the FMO thin film during lithiation. (c) Voltage curve and (d) main peak position variation of the film during delithiation. The (dis)charge current density was 1.0 μA cm^−2^ (∼0.3C).

Fig. 3a presents the voltage curve of the FMO thin film during lithiation with the charging current density of 1.0 μA cm^−2^ (∼0.3C). The horizontal axis corresponds to the voltage against the reference FMO electrode, while the vertical axis denotes the areal capacity of the FMO thin film. The charge capacity of approximately 3.2 μAh cm^−2^ agreed well with the theoretical capacity of FMO, suggesting that the FMO thin film was almost fully lithiated during charging. As shown in this figure, the voltage of the FMO thin film gradually changed with lithiation, and no distinct potential plateaus were confirmed, in contrast to the lithiation in liquid electrolytes.^37,38^Fig. 3b shows the evolution of the main peak of the FMO thin film during lithiation. Throughout the lithiation process, essentially a single FMO main peak was observed. The position of the FMO main peak gradually shifted towards lower *Q* values during lithiation, finally reaching the peak position of Li_2_FMO. Fig. 3c and d show the voltage curve and peak evolution of the FMO thin film during delithiation with the same (dis)charging rate, respectively. The evolutions of the voltage and main peak of the FMO thin film during delithiation were generally symmetric to those during lithiation. During delithiation, the voltage gradually increased, and the main peak shifted progressively to higher *Q* values, eventually returning to nearly the same position as before lithiation. As described above, the FMO on a solid electrolyte did not exhibit the reported peak variation characteristic of a two-phase reaction, *i.e.*, a gradual decrease/increase in FMO peak intensity at a fixed position and a corresponding increase/decrease in Li_2_FMO peak intensity at another fixed position, during lithiation/delithiation (see also ESI 3†).^37,38^ The lithiation/delithiation behaviour of the FMO thin film on the solid electrolyte was thus confirmed to differ from that in liquid electrolytes. The observed monotonous shift of a FMO peak upon lithiation/delithiation suggests that the lithiation/delithiation of FMO on a solid electrolyte proceeded *via* a solid-solution reaction rather than a two-phase reaction. On the other hand, focusing on the peak shape in the middle stage of lithiation (∼1.0–2.6 μAh cm^−2^), a small shoulder appeared on the left side of the main peak, temporarily broadening the peak width. Subsequently, the intensity of the right side of the peak decreased, rendering the peak sharper again. Similar to the case of lithiation, the transient peak shape variation was also observed during delithiation within the same capacity range. Such peak shape variations may be an indication of the two-phase reaction within the narrow capacity range in the middle stages of lithiation/delithiation. To summarize, our results indicate that the lithiation/delithiation behaviour of the FMO thin film on a solid electrolyte was markedly different from that in a liquid electrolyte. Considering the observed variations in the FMO main peak, it can be inferred that the lithiation/delithiation reaction of FMO on a solid electrolyte has a larger solid-solution reaction region and a more limited two-phase reaction region in the intermediate Li composition range, compared to that in a liquid electrolyte. These results suggest that the (dis)charge mechanism of FMO was modified due to some factors upon the battery solidification. Then, the next question is what factor altered the lithiation/delithiation behaviour of FMO on the solid electrolyte. The potential factors that influence the (dis)charge behaviour can be broadly classified into two groups, namely, thermodynamic factors and kinetic ones. If a kinetic factor is responsible for the observed variation in the lithiation/delithiation behaviour of FMO on the solid electrolyte, the lithiation/delithiation behaviour will differ depending on (dis)charge currents. Therefore, we next examined the (dis)charge rate dependence of the lithiation/delithiation behaviour of the FMO thin film to assess the impact of kinetic factors.

### (Dis)charge rate dependence of lithiation/delithiation behaviour of the FMO thin film

**Fig. 4:**
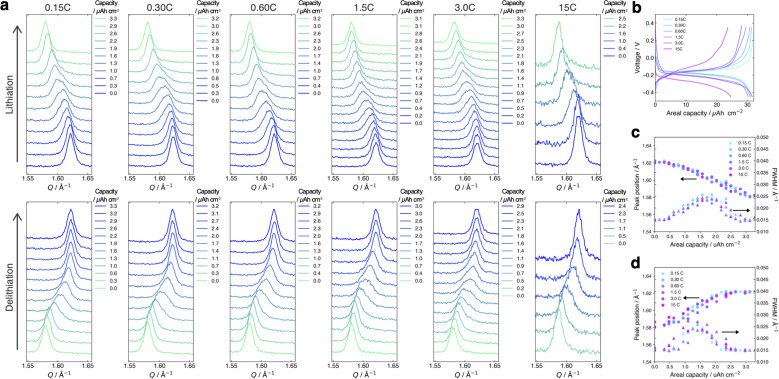
(a) Evolution of the main peak of the FMO thin film and (b) voltage curves of the FMO thin film during lithiation/delithiation at various (dis)charge rates ranging from 0.15C to 15C. Evolutions of the peak position and FWHM of the FMO main peak during (c) lithiation and (d) delithiation at various (dis)charge rates.

**Fig. 5:**
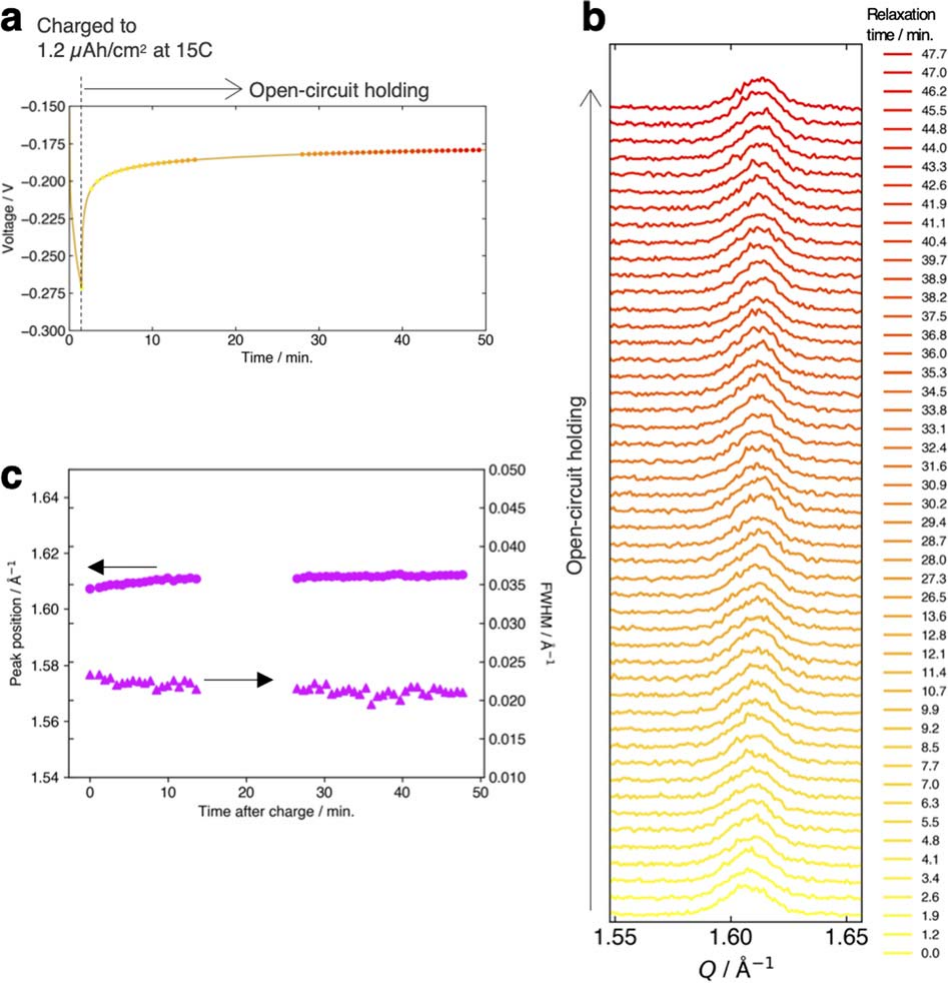
(a) Voltage variation of the FMO thin film during 15C lithiation and successive open-circuit holding. Evolution of (b) the main peak of the FMO thin film and (c) its peak position and FWHM during the open-circuit holding after the 15C lithiation.

Fig. 4a and b present the evolution of the FMO main peak during lithiation/delithiation and the voltage curve of the thin film, respectively, at various (dis)charge rates ranging from 0.15C to 15C (the (dis)charge curves with the voltage referenced against the LCO thin film are shown in ESI 4†). Due to an equipment malfunction during the measurement, the latter half of the charging data and the first half of the discharging data were lost for the voltage curve at 0.15C. While the FMO thin film showed a degraded (dis)charge capacity of 74–78% of the theoretical capacity at a rate of 15C, it exhibited a (dis)charge capacity close to the theoretical value at lower rates. Regardless of the (dis)charge rate, the peak position changed monotonically with lithiation/delithiation in a broad capacity range, and the transient broadening of the peak widths was observed in the middle stage of lithiation/delithiation. As shown in these figures, despite varying the (dis)charge current by two orders of magnitude, the peak evolution behavior during lithiation/delithiation appeared to be nearly independent of the (dis)charge current. To quantitatively track the variations of the position and width of the FMO main peak during lithiation/delithiation at each rate, we fitted the main peak with a Gaussian function and estimated its peak position and full width at half maximum (FWHM). Fig. 4c and d illustrate the changes in the position and FWHM of the FMO thin film during lithiation and delithiation, respectively, at various (dis)charge rates. At all (dis)charging rates, the peak position monotonically decreased and increased during lithiation and delithiation, respectively, with a temporary increase in FWHM in the intermediate capacity range. The differences in the peak position and FWHM between various (dis)charge rates were insignificant. Additionally, no systematic changes in the variation of the peak characteristics were observed with respect to the (dis)charge rate. These results suggest that the lithiation/delithiation behaviour of FMO on a solid electrolyte is not rate-dependent, and thus is not governed by kinetic factors. To further investigate the influence of kinetic factors, we examined the evolution of the FMO peak during the open-circuit period following high-rate lithiation. As depicted in Fig. 5a, the FMO thin film was lithiated at 15C to approximately 1.2 μAh cm^−2^, a capacity at which distinct peak separation was observed with the FMO in the liquid electrolyte,^37,38^ and then held under open-circuit conditions. If the lithiation/delithiation behaviour of FMO on a solid electrolyte is altered by a kinetic factor during lithiation, FMO should gradually transform to a thermodynamically equilibrated state, *i.e.*, a two-phase coexistence of FMO and Li_2_FMO, during open-circuit holding. Accordingly, the single main peak of FMO should gradually split into two peaks corresponding to the Li-rich and Li-poor phases. As illustrated in Fig. 5b, however, the FMO peak did not exhibit significant changes during the open-circuit period. Fig. 5c presents corresponding evolutions of the peak position and FWHM of the FMO peak. Although a slight shift in peak position towards higher *Q* values and a minor decrease in FWHM were observed within around 10 minutes of the open-circuit period, these changes were inconsistent with those expected when FMO approaches a two-phase state. From these results, it is strongly suggested that the observed changes in the lithiation/delithiation behaviour of FMO on the solid electrolyte are not caused by kinetic factors.

### Discussion on the thermodynamic alteration mechanism of the lithiation/delithiation behaviour of FMO on a solid electrolyte

If the influence of kinetic factors is negligible, the changes in the lithiation/delithiation behaviour of FMO on the solid electrolyte can be attributed to thermodynamic factors. A primary thermodynamic factor potentially influencing the lithiation/delithiation behaviour of FMO on a solid electrolyte is the stress arising from the constraint of the volume change of the FMO thin film by the solid electrolyte substrate during lithiation/delithiation. FMO is known to exhibit a large lattice constant change of up to several percent upon lithiation/delithiation.^37^ Assuming an elastic modulus of approximately 100 GPa for FMO for simplicity, the constraint of this lattice deformation is roughly estimated to generate a stress ranging from several hundred MPa to a few GPa within the FMO thin film. Such large stresses may alter the lithiation/delithiation mechanism of FMO. Indeed, stress is reported to alter the Li chemical potential of each phase in the two-phase cathode material LiFePO_4_.^32^ Furthermore, it has been reported that interfacial strain between the substrate and thin film can modify the lithiation mechanism of Fe_3_O_4_ thin films, a conversion-type anode material.^30^ In the following, therefore, we discussed whether stress can thermodynamically alter the lithiation/delithiation behaviour of FMO. More specifically, we discussed if stress can thermodynamically expand the solid solution region and contract the two-phase region of FMO as suggested by our XRD measurements. Furthermore, we discussed what properties of FMO rendered its lithiation/delithiation mechanism stress-dependent.

**Fig. 6:**
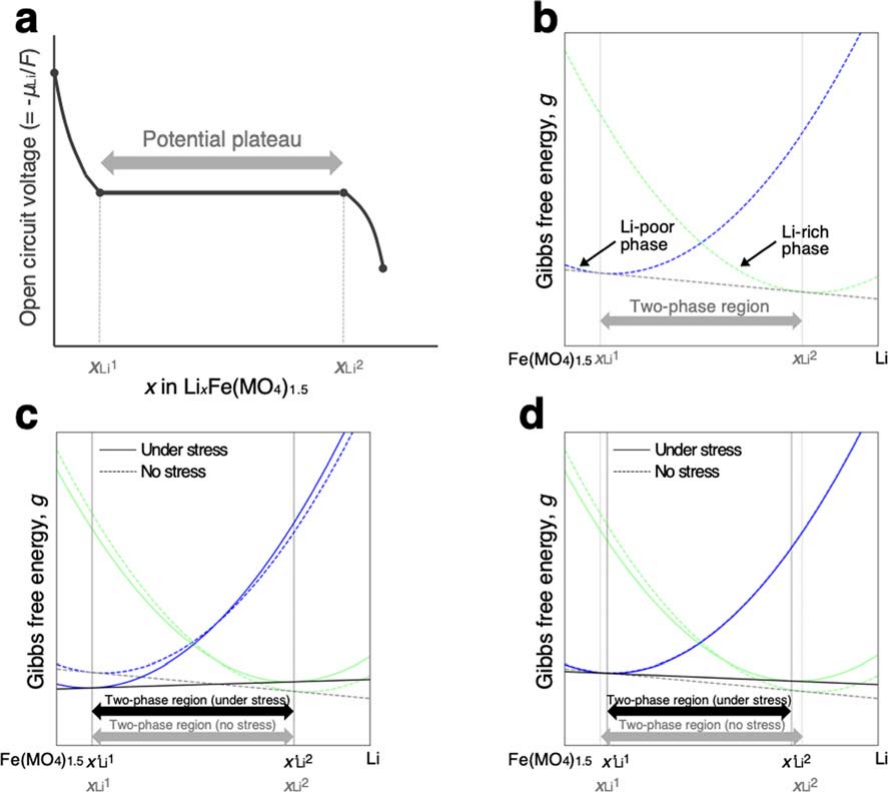
(a) Schematic of open circuit voltage of FMO as a function of its Li content. (b)Schematic of the Gibbs free energy curves of Li-poor and Li-rich phases of FMO. Schematic of the variation of the Gibbs free energy curve of each phase based on the eqn (5) under the assumption of (c)equal and (d) different *α*^chem^ for each phase.

As schematically illustrated in Fig. 6a, the two-phase region of FMO corresponds to the Li compositional range where the Li chemical potentials (*μ*_Li_) of the Li-rich and Li-poor phases are balanced, resulting in a potential plateau in the open-circuit voltage curve. Thermodynamically, this region corresponds to the Li compositional range between the points of tangency of the common tangent line with the Gibbs free energy curves of each phase as illustrated in Fig. 6b. Therefore, we here examined how stress altered the Gibbs free energy curve of each phase in FMO and the common tangent line. Due to the lack of necessary parameters to quantitatively describe the Gibbs free energy curve of each phase and the elastic constants of FMO, we focused on a qualitative discussion of the influence of stress on the lithiation/delithiation behaviour, based on the general thermodynamic formula of the Gibbs free energy curves. The energy change of both Li-poor and Li-rich phases of FMO under strain can be expressed using the Helmholtz free energy,1
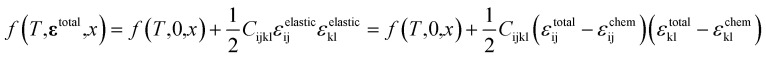
where *f*(*T*,**ε**^total^,*x*) and *f*(*T*,0,*x*) represent the Helmholtz free energy per unit volume of each phase under strain and without strain, respectively. The third term denotes the elastic strain energy, where *C*_ijkl_ is the elastic constant and *ε*_ij_^elastic^ is an element of the elastic strain tensor, **ε**^elastic^. **ε**^total^ is the total strain tensor of each phase, which is composed of the elastic strain tensor and the chemo-mechanical strain tensor, **ε**^chem^:

2 **ε**^total^ = **ε**^elastic^ + **ε**^chem^

Using this equation, the Helmholtz free energy under stress can also be expressed as shown in the second line of eqn (1). By performing a Legendre transformation of this equation with respect to strain, the Gibbs free energy per unit volume of each phase under stress can be obtained as follows:3
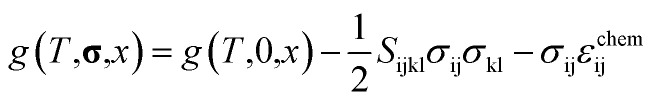
where *g*(*T*,**σ**,*x*)and *g*(*T*,0,*x*) represent the Gibbs free energy of each phase under stress and without stress, respectively. The second and third terms on the right-hand side denote the contribution of stress, where *S*_ijkl_ is the elastic compliance and **σ** is the stress tensor. Assuming Vegard's law, the chemo-mechanical strain of each phase is expressed as follows:4
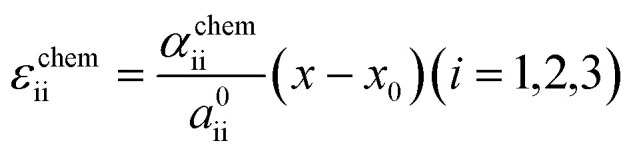
where *α*_ii_ denotes the chemical expansion coefficient, which represents the slope of the lattice constant of each direction (*i* = 1, 2, 3 corresponds to *a*, *b*, *c*-axis, respectively) with respect to the Li content in FMO (*x* in Li_*x*_Fe(MO_4_)_1.5_). *x*_0_ and *a*^0^_ii_ are the reference Li content and the reference lattice constant of each axis, respectively. According to Yue *et al.*, the lattice constants of FMO along the *a*, *b*, and *c* axes exhibit anisotropic changes upon lithiation, with maximum variations of approximately −0.7, 2.0, and −0.3%, respectively.^37^ For simplicity, we neglect the chemo-mechanical strain along the *a*-axis, because our FMO thin film exhibited a localized *a*-axis orientation and are less likely to be constrained in this direction. Furthermore, we neglect the chemo-mechanical strain along the *c*-axis since it is much smaller compared to that along the *b*-axis. Therefore, we consider only the chemo-mechanical strain along the *b*-axis (*i* = 2). Using eqn (4) and under aforementioned assumptions, the Gibbs free energy can be expressed as follows:5
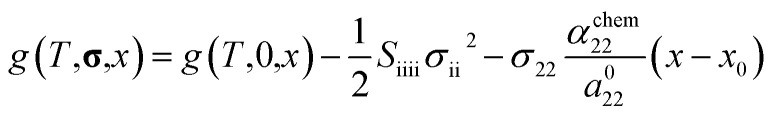


To estimate the variation of the Gibbs free energy curve under stress based on this equation, we first make a simplifying assumption that *α*^chem^ along the *b*-axis, *α*^chem^_22_, is equal for each phase. In this case, the Gibbs free energy curve of each phase varies under compressive stress (*σ*_22_ < 0) as shown in Fig. 6c. Here, the reference Li content was set to *x* = 0.5. Moreover, we assumed that the elastic compliance of each FMO phase was equal and independent of the Li content for simplicity. Under these assumptions, the second term in eqn (5) decreases the Gibbs free energy curve of each phase by a constant value. In addition to this, the third term modifies the free energy curves by adding a linear term to each curve. The common tangent lines of the free energy curves under stress and without stress are represented by the black solid and grey dashed lines in Fig. 6c, respectively. As shown in this figure, although the free energy curves of both phases and the common tangent line were varied under stress, the positions of the contact points between the curves and the common tangent line, *i.e.*, the upper and lower limits of the two-phase coexistence region, remain unchanged in the case of equal *α*^chem^ for each phase. This result is natural, as equal *α*^chem^ for both phases simply leads to the addition of an identical linear term to the curves of both phases. From these discussions, we can conclude that if *α*^chem^ is equal for both phases, the stress will not thermodynamically change the two-phase region, regardless of its magnitude. Next, we consider the case where the *α*^chem^ differs for each phase, which is more likely to be a case of FMO. It is reported that the change in the lattice constant in the *b*-axis during lithiation is smaller for the Li-poor phase compared to the Li-rich phase,^37^ indicating that the *α*^chem^ along the *b*-axis is smaller for the Li-poor phase than the Li-rich phase. This is also indirectly supported by our XRD measurements of the FMO thin film. As shown in Fig. 4b and c, our results suggest that regions with lower Li content exhibit smaller changes in peak position, *i.e.*, smaller lattice constant variations, during lithiation/delithiation compared to regions with higher Li content. The variations of the Gibbs free energy curves in this case are illustrated in Fig. 6d, where we assumed that the *α*^chem^ of the Li-poor phase is considerably smaller than that of the Li-rich phase. The common tangent lines under stress and without stress under these conditions are represented by the solid black and grey dashed lines, respectively. In this case, the two-phase region becomes narrower under stress, because the different *α*^chem^ for both phases leads to the addition of a different linear term to each phase. These discussions qualitatively reveal that for the stress to thermodynamically affect the two-phase region of electrode materials including FMO, the *α*^chem^ of each phase must differ. Moreover, the magnitude of the stress-induced variation of the two-phase region is dependent on the difference in the *α*^chem^ between the phases, rather than the absolute values of *α*^chem^ for each phase, with larger differences resulting in more pronounced variation in the two-phase region. Based on the above discussions, it is suggested that the lithiation/delithiation mechanism of FMO on a solid electrolyte could be varied due to the stress generated by the substrate constraints because the *α*^chem^ of the Li-rich and Li-poor phases of FMO were different. As shown in the above discussion, when applying two-phase electrode materials including FMO to ASSLIBs, it is important to consider not only the difference in lattice constants between the phases, which determines the magnitude of the chemo-mechanical stress, but also the disparity in *α*^chem^, as it governs the influence of stress on the (dis)charge mechanism.

## Conclusions

We examined the lithiation/delithiation behavior of FMO thin films deposited on solid electrolytes using *operando* synchrotron XRD to gain insights into the influence of the battery solidification on the (dis)charge mechanism of electrode materials. During (dis)charge, the main XRD peak of FMO on the solid electrolyte exhibited a monotonic shift over a wide capacity range, accompanied by a temporary increase in peak width in the middle stage of (dis)charge, which was markedly different from the peak evolution associated with the typical two-phase reaction of FMO in liquid electrolytes. These results suggest that FMO on solid electrolytes exhibits an expanded solid-solution reaction region and a limited two-phase reaction region compared to FMO in liquid electrolytes. The above-described lithiation/delithiation behavior remained largely unchanged with varying (dis)charge rates, as well as under open-circuit holding after fast lithiation, suggesting that the altered (dis)charge mechanism of FMO on solid electrolytes is governed by thermodynamic factors rather than kinetic ones. Considering the stress-induced variations in the Gibbs free energy curves of each phase, it was qualitatively demonstrated that the external stress, which is potentially caused by the constraint of chemo-mechanical expansion of FMO by a solid electrolyte, can narrow the two-phase reaction region. Moreover, it was shown that for the stress to thermodynamically alter the two-phase region, the *α*^chem^ of each phase of the two-phase material must differ. As described above, when the electrode materials are exposed to large stresses due to LIB solidification, their properties can significantly deviate from that in liquid electrolytes. Therefore, when designing ASSLIBs, it is important to consider the unique issues associated with the LIB solidification, including stresses, to ensure that the electrode materials can sufficiently exhibit their intrinsic performance.

## Author contributions

Yuta Kimura: conceptualization, funding acquisition, project administration, supervision, data curation, investigation, writing – original draft, writing – review & editing. Takashi Nakamura: investigation and data curation. Shintaro Kobayashi: investigation, resources, methodology. Shogo Kawaguchi: investigation, resources, methodology. Koji Ohara: investigation, resources, methodology. Yasuhiro Suzuki: investigation, resources. Yasutoshi Iriyama: funding acquisition, resources, conceptualization, and supervision. Koji Amezawa: funding acquisition, resources, conceptualization, and supervision.

## Conflicts of interest

There are no conflicts to declare.

## Supplementary Material

RA-014-D4RA03058C-s001
