# Supplementary material for: Modifications of the charge–discharge behaviour of Fe2(MoO4)3 in all-solid-state lithium-ion batteries
Source: RSC Adv. 2024 Jun 6;14(26):18109–16. doi: 10.1039/d4ra03058c (PMC11154690; doi:10.1039/d4ra03058c)
Supplement: RA-014-D4RA03058C-s001 [file RA-014-D4RA03058C-s001.pdf]

Electronic Supplementary Material (ESI) for RSC Advances.
This journal is © The Royal Society of Chemistry 2024

Supplementary information

Modifications of the Charge-Discharge Behaviour of $Fe_2(MoO_4)_3$ in All-Solid-State Lithium-Ion Batteries

Yuta Kimura,*[a] Shintaro Kobayashi,[b] Shogo Kawaguchi,[b] Koji Ohara,[b,c] Yasuhiro Suzuki,[d] Takashi Nakamura,[a] Yasutoshi Iriyama,[d] and Koji Amezawa [a]

[a] Institute of Multidisciplinary Research for Advanced Materials, Tohoku University, 2-1-1, Katahira, Aoba-Ku, Sendai, 980-8577, Japan. E-mail: yuta.kimura.b2@tohoku.ac.jp

[b] Japan Synchrotron Radiation Research Institute, 1-1-1, Koto, Sayo-cho Sayo-gun, Hyogo, 679-5198, Japan

[c] Faculty of Materials for Energy, Shimane University,1060, Nishikawatsu-cho, Matsue, Shimane, 690-0823, Japan

[d] Graduate School of Engineering, Nagoya University, Furo, Chikusa, Nagoya, Aichi 464–8603, Japan

*Corresponding author: yuta.kimura.b2@tohoku.ac.jp

Supplementary information 1. Characterizations of the FMO thin film

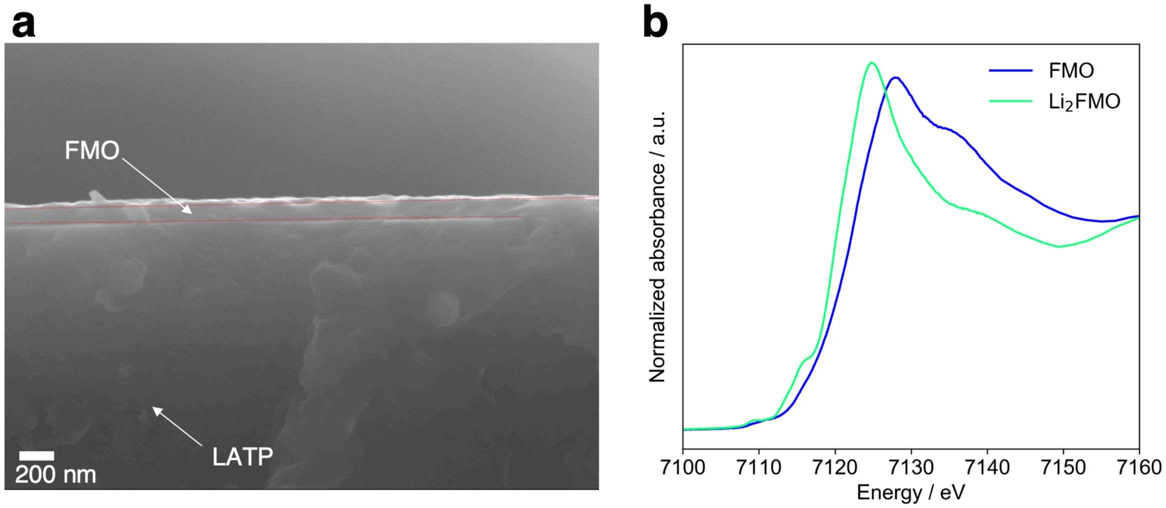

Fig. S1. (a) Cross sectional SEM image of the FMO thin film. The film thickness was estimated to be approximately 100 µm, which was corresponded with the values measured using a stylus surface profilometer. (b) X-ray absorption near edge structure (XANES) spectra of the FMO thin film before and after lithiation. The XANES measurements were performed by a partial fluorescence yield method at the beamline BL37XU, SPring-8, Japan. The observed changes in the spectra agreed with the literature[1,2]. Other analysis results, such as Raman spectroscopy, optical microscopy observations, and conventional electrochemical evaluations such as linear sweep voltammograms, have been presented in our previous literature[3].

Supplementary information 2. XRD pattern of the FMO thin film after completing all measurements

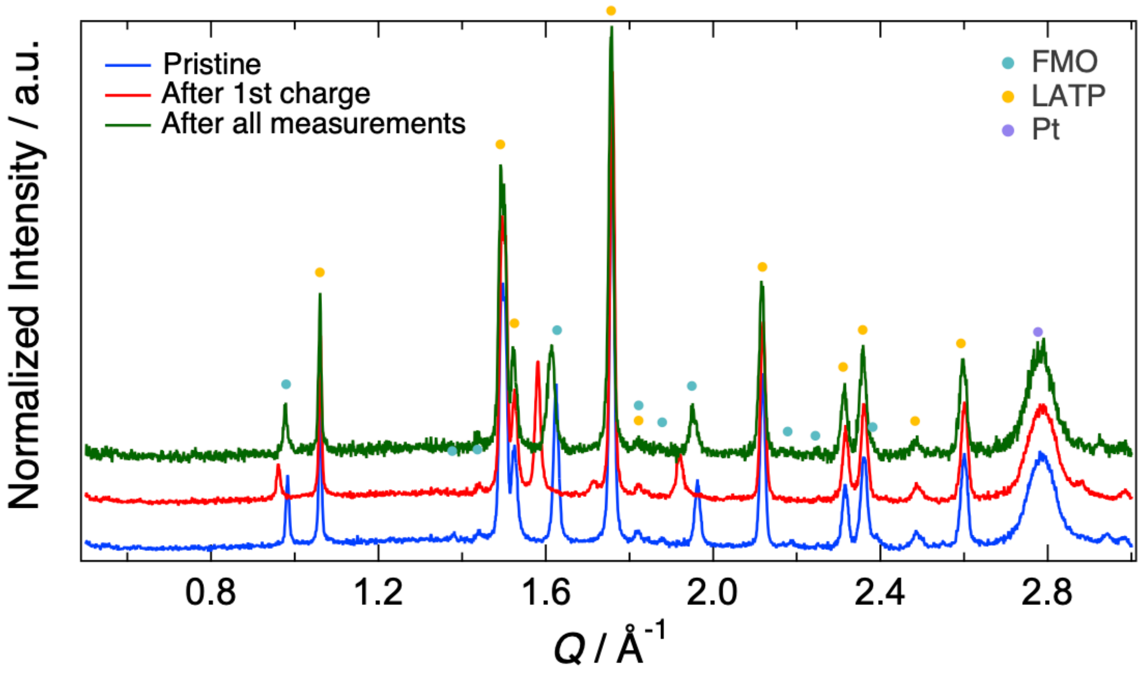

Fig. S2 XRD patterns of the pristine FMO thin film (blue), that after 1st charge (green) and after completing all measurements (red). The exposure time for the XRD measurements of the pristine FMO thin film and that after the first charge was 60 seconds, while the exposure time for the XRD measurement after all measurements was 10 seconds. The final XRD measurement was performed after charging the FMO to half of its theoretical capacity (Fig. 5 in the main text); therefore, the FMO peaks after all measurements are generally located between those of the pristine and charged states. Due to the shorter exposure time, the signal-to-noise ratio of the XRD pattern after all measurements was lower. However, no significant changes were observed in the XRD pattern, suggesting that the FMO remained stable after all measurements.

Supplementary information 3. XRD patterns of FMO during lithiation/delithiation in liquid electrolytes reported in previous works

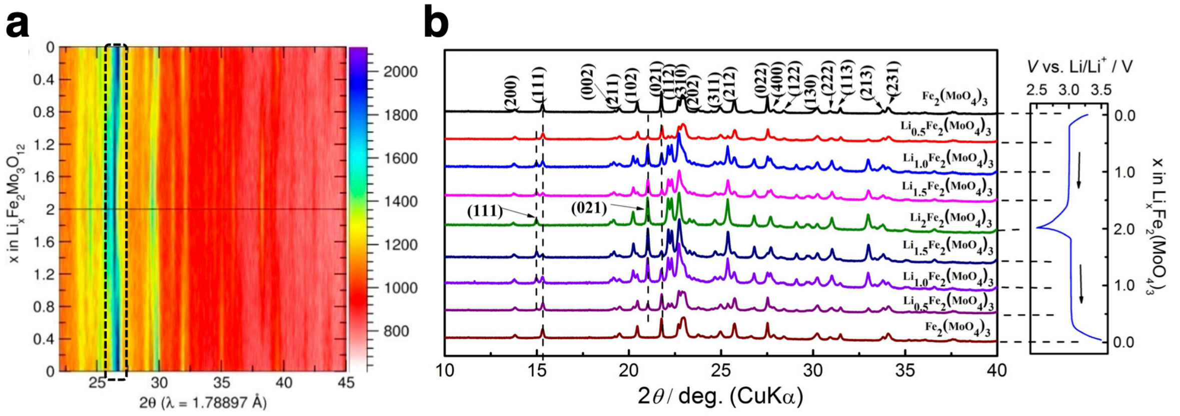

Fig. S3. XRD patterns of FMO during lithiation/delithiation in liquid electrolytes reported by (a) Zhou *et al.*[4] and (b) Yue *et al.*[5], respectively. Fig S3 (a) is adapted with permission from S. Zhou *et al.*, *Chem. Mater.*, 2016, **28**, 4492–4500, Copyright 2024 American Chemical Society. As indicated by the black lines, the two XRD patterns clearly demonstrate that FMO undergoes (de)lithiation via a two-phase reaction in liquid electrolytes.

Supplementary information 4. (Dis)charge curves of the FMO thin film with the voltage referenced against the LCO thin film

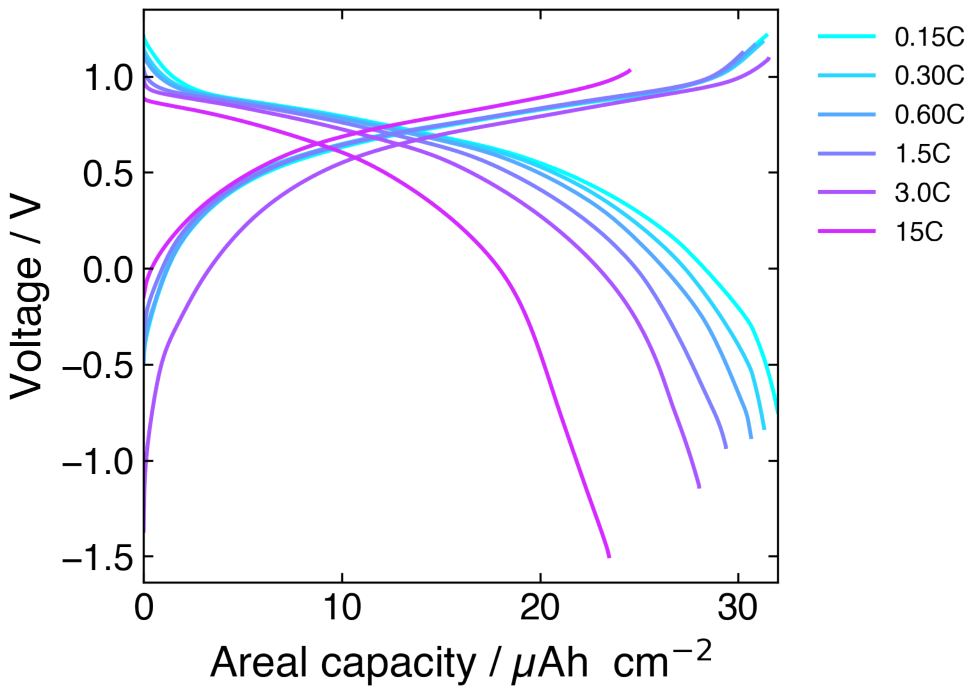

Fig. S4 (Dis)charge curves of the FMO thin film at various (dis)charging rates with the voltage referenced against the LCO thin film.

References

1 J. Shirakawa, M. Nakayama, M. Wakihara and Y. Uchimoto, *J. Phys. Chem. B*, 2007, **111**, 1424–1430.

2 N. Ishiguro, T. Totsuka, H. Uematsu, O. Sekizawa, K. Yamamoto, Y. Iriyama and Y. Takahashi, *ACS Appl. Energy Mater.*, 2023, **6**, 8306–8315.

3 Y. Kee, Y. Suzuki, N. Ishigaki, M. Motoyama, Y. Kimura, K. Amezawa and Y. Iriyama, *Electrochem. commun.*, 2021, **130**, 107108.

4 S. Zhou, G. Barim, B. J. Morgan, B. C. Melot and R. L. Brutchey, *Chem. Mater.*, 2016, **28**, 4492–4500.

5 J.-L. Yue, Y.-N. Zhou, S.-Q. Shi, Z. Shadike, X.-Q. Huang, J. Luo, Z.-Z. Yang, H. Li, L. Gu, X.-Q. Yang and Z.-W. Fu, *Sci. Rep.*, 2015, **5**, 8810.
